# Features of virtual reality impact effectiveness of VR pain alleviation therapeutics in pediatric burn patients: A randomized clinical trial

**DOI:** 10.1371/journal.pdig.0000440

**Published:** 2024-01-25

**Authors:** Soumil Jain, Megan Armstrong, John Luna, Rajan K. Thakkar, Renata Fabia, Jonathan I. Groner, Dana Noffsinger, Ai Ni, Eric Nelson, Henry Xiang

**Affiliations:** 1 College of Medicine, The Ohio State University, Columbus, Ohio, United States of America; 2 Center for Pediatric Trauma Research, The Abigail Wexner Research Institute at Nationwide Children’s Hospital, Columbus, Ohio, United States of America; 3 Center for Injury Research and Policy, The Abigail Wexner Research Institute at Nationwide Children’s Hospital, Columbus, Ohio, United States of America; 4 IT Research and Innovation, The Abigail Wexner Research Institute, Nationwide Children’s Hospital, Columbus, Ohio, United States of America; 5 Trauma and Burn Program, Nationwide Children’s Hospital, Columbus, Ohio, United States of America; 6 Division of Biostatistics, The Ohio State University College of Public Health, Columbus, Ohio, United States of America; 7 Center for Biobehavioral Health, The Abigail Wexner Research Institute at Nationwide Children’s Hospital, Columbus, Ohio, United States of America; Polytechnic Institute of Porto: Instituto Politecnico do Porto, PORTUGAL

## Abstract

Key features of virtual reality (VR) that impact the effectiveness of pain reduction remain unknown. We hypothesized that specific features of the VR experience significantly impact VR’s effectiveness in reducing pain during pediatric burn dressing care. Our randomized controlled trial included children 6 to 17 years (inclusive) who were treated in the outpatient clinic of an American Burn Association–verified pediatric burn center. Participants were randomly assigned (1:1:1) to active VR (playing the VR), passive VR (immersed in the same VR environment without interactions), or standard-of-care. On a scale from 0 to 100, participants rated overall pain (primary outcome) and features of the VR experience (game realism, fun, and engagement). Path analysis assessed the interrelationships among these VR key features and their impact on self-reported pain scores. From December 2016 to January 2019, a total of 412 patients were screened for eligibility, and 90 were randomly assigned (31 in the active VR group, 30 in the passive VR group, and 29 in the standard-of-care group). The current study only included those in the VR groups. The difference in median scores of VR features was not statistically significant between the active (realism, 77.5 [IQR: 50–100]; fun, 100 [IQR: 81–100]; engagement, 90 [IQR: 70–100]) and passive (realism, 72 [IQR: 29–99]; fun, 93.5 [IQR: 68–100]; engagement, 95 [IQR: 50–100]) VR distraction types. VR engagement had a significant direct (-0.39) and total (-0.44) effect on self-reported pain score (p<0.05). Key VR features significantly impact its effectiveness in pain reduction. The path model suggested an analgesic mechanism beyond distraction. Differences in VR feature scores partly explain active VR’s more significant analgesic effect than passive VR.

**Trial Registration:** ClinicalTrials.gov Identifier: NCT04544631.

## Author summary

In this randomized controlled trial of 90 pediatric burn patients aged 6–17 years (inclusive) who received burn dressing changes at a pediatric medical center, three key features of virtual reality (VR) (game realism, fun, and engagement) significantly influence self-reported pain scores during burn dressing changes. These findings provide insight into the potential mechanisms through which VR affects pain perception, supporting distraction as a key mechanism of VR analgesia and highlighting a potential neurophysiological mechanism beyond mere distraction. Future studies should employ longitudinal designs, develop objective measures of VR features, develop methods to manipulate VR features, consider clear and specific terminology to describe digital experiences, and further test key features of VR games that could effectively impact the VR pain alleviation therapeutics during a variety of medical procedures.

## Introduction

Pain–an unpleasant sensory and emotional experience associated with or resembling actual or potential tissue damage–inflicts a substantial global burden on society [1,2]. In pediatric healthcare, pain’s prevalence is alarmingly high, with a study at a U.S. children’s hospital highlighting that approximately 75% of hospitalized children experience pain, and up to 30% endure severe pain [3]. According to a study of eight Canadian pediatric hospitals, most hospitalized children undergo at least one painful procedure [4]. Therefore, effective pain management is an essential component of pediatric health care. Furthermore, the need for non-pharmacologic approaches to pain management has become increasingly evident due to the harmful side effects of opioid analgesics [5]. In this context, virtual reality (VR) has emerged as a promising noninvasive, nonpharmacologic, and effective alternative and/or addition for pain management. VR has effectively reduced pain during medical procedures such as burn care, dental care, and needle-related interventions [6–8].

As VR technology is increasingly accessible, users may become less engaged with the novelty of this technology, and its ability to engage patients may decline over time [9]. Therefore, focusing on designing more impactful VR interventions is essential. Previous studies have demonstrated efforts to improve VR effectiveness in pain alleviation by increasing interactivity and feeling of presence [10–12]. Active VR, where users interact with a VR environment, has a superior analgesic effect over passive VR, where users observe a VR environment [8]. Furthermore, higher-tech VR hardware increases VR’s sense of presence and analgesic effectiveness [11,12]. However, to maximize VR’s analgesic potential, we must measure the impact of specific features of the VR experience, such as the degree of VR game realism, fun, and engagement, on pain reduction.

Various features of the VR experience have been proposed as key areas of interest for designers of clinical VR applications, including presence, interactivity, social interaction, embodiment, immersion, and agency [9,13,14]. A holistic understanding of these features, alongside other pertinent aspects of VR, may lead to the design of more effective VR experiences for healthcare [9]. Despite numerous literature reviews highlighting the clinical relevance of VR features, the impact of VR features has yet to be thoroughly evaluated in a randomized controlled trial (RCT). However, VR features’ specific effects on pain, their interrelationships, and the potential mechanisms through which they exert their effects are not yet well-defined.

In our previous publication based on data from a RCT among 6–17 years old burn patients [8], our VR Pain Alleviation Therapeutics (VR-PAT) proved to be an effective pain management, and patients rated the VR-PAT features: game realism, fun, and engagement using a numeric scale of 0–100. Using burn dressing as an example of medical procedures in which VR could be an effective non-pharmacologic pain alleviation, the purpose of this study was to evaluate the degree to which each of VR-PAT’s three key features impacted the effectiveness of pain reduction and their interrelationships, and discussed the potential mechanisms by which they operate. We hypothesized a path model of these VR features and their relation to clinical self-reported pain scores by patients.

Although we are using burn dressing change as a model condition to assess VR engagement features, we note that managing pain during burn dressing change is a particularly important specific context in which to implement VR methods. Dressing changes are a necessary component of burn treatment and management of pain is essential. Our previous study demonstrated that VR is an effective means of managing pain even in pediatric patients with 2^nd^ degree burns covering greater than 1% of surface area. Thus while the primary purpose of this study was to understand features of VR engagement across contexts it is clearly a key aspect that is specific to burn care management as well.

## Methods

### Study design and participants

We conducted a randomized controlled trial (RCT) at an American Burn Association-verified U.S. pediatric burn center between December 2016 and January 2019. Eligible participants were children 6 to 17 years (inclusive) with a burn injury who were treated in the outpatient burn clinic and spoke English as their primary language. Patients were excluded if they had (1) a severe burn on the face or head that prevented VR use; (2) cognitive or motor impairment that prevented administering study measures; (3) visual or hearing impairments that prevented VR interaction; or (4) no legal guardian present to give consent. A medical record review identified potential participants, and a trained researcher in the outpatient clinic approached eligible participants before their appointment. Following informed consent, the patient’s medical record provided sex (male or female).

### Ethics statement

The Institutional Review Board at Nationwide Children’s Hospital reviewed and approved this study. One legal guardian provided formal written informed consent, and children nine years and older signed written assent. Our previous publication published the study protocol as a supplemental file [8]. This trial is registered with ClinicalTrials.gov, identifier: NCT04544631.

### Randomization and masking

Participants were randomly assigned to active VR, passive VR (described below), or standard of care control group (1:1:1 ratio, stratified by sex). Because this study specifically focuses on features of the VR experience, the results from the standard-of-care group have been excluded. The results from this clinical trial comparing VR interventions with the standard of care have been previously published [8].

One researcher enrolled participants, implemented computerized randomization, and administered the VR intervention. Recruiters were not aware of the randomization scheme hosted within REDCap. This researcher and nurses providing burn care were masked to group assignment until immediately before intervention. A second researcher, masked to group assignment, administered a postintervention survey about child perceived pain and VR experience. Researchers analyzing the data were not masked to group assignment. Due to the nature of the intervention, participants were not masked to group assignment.

### Procedures

A detailed description of the trial procedures has been published and is provided in the study protocol [8]. Following informed consent, participants were asked to fill out a pre-intervention survey about their pain expectations, desire for a fun activity during burn care, anxiety, and pain medication use before burn care. The patient’s electronic medical record provided demographics and burn injury variables. Immediately before the burn dressing change, a trained researcher administered the VR pain alleviation therapeutic (VR-PAT) to those in the VR groups, which consists of a lightweight, low-cost VR viewer and an engaging VR game played on a smartphone. The Research Information Solutions and Innovation Department of Nationwide Children’s Hospital developed VR-PAT.

The passive VR participants were immersed in the same VR environment as the active VR group but did not interact with the VR game.

Participants assigned to active VR played a VR game titled Virtual River Cruise, in which the player experiences a first-person perspective floating on a boat beside an otter. As they journey down a river, snow-emitting statues appear along their path. The statue emits snow and disappears when the child tilts their head to aim at the figure correctly. To increase engagement, a thermometer placed on the boat shows decreasing temperatures as the statues release more snowflakes, and a scoreboard beside the thermometer displays the number of activated statues. Additionally, as the temperature drops, snow starts piling up on the boat and its surroundings, providing a potentially cooling experience for the burn patients. A pair of headphones provides surrounding and directionally adaptive audio effects to match the game’s progress and the direction in which a child’s head turns, further enhancing the immersive experience. Children play the VR game solely by tilting their heads, minimizing interference with the dressing change.

**Fig 1:**
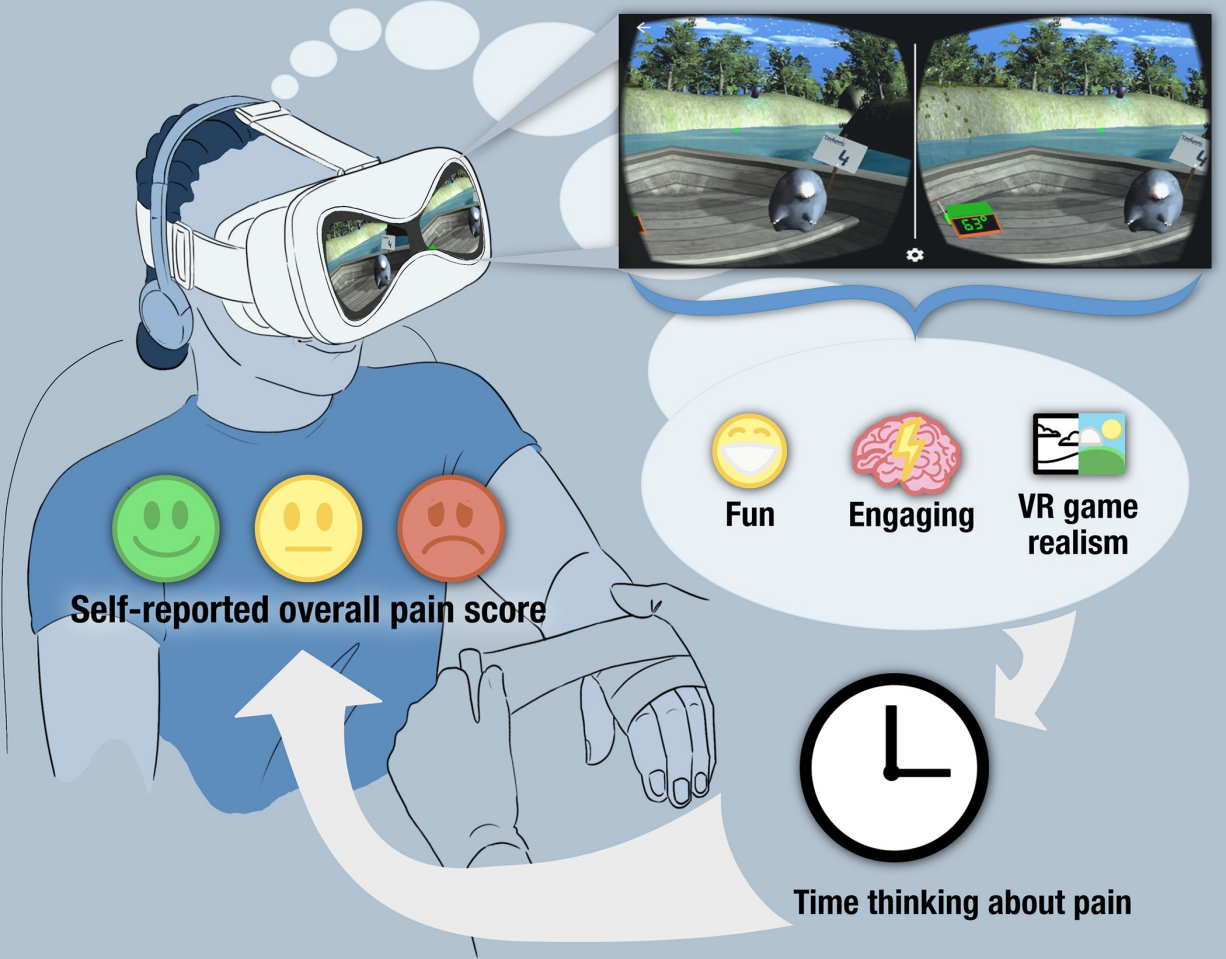
Conceptual model of the VR pain alleviation therapeutic study. Legend—A postulated conceptual model for VR pain reduction effect during burn dressing changes. Key features of the patient VR experience (VR game realism, fun, and engagement) significantly impact the patient’s self-reported pain score via the intermediate variable time thinking about pain during the burn dressing changes (distraction theory, indirect effect) and modulate the brain “pain matrix” areas (neurophysiologic mechanism theory, direct effect).

After the intervention, children completed a post-intervention survey in which they self-reported their pain, time spent thinking about pain using a numerical scale ranging from 0–100, with 0 indicating not thinking about pain at all and 100 indicating thinking about pain the whole time, the degree of realism experienced in the VR environment, the degree of fun associated with playing the VR game, and the perceived engagement level with VR distraction during the procedure (see Fig 1 for the conceptual model of this study).

### Outcomes

The primary outcome was patient-perceived pain, rated on a 0–100 visual analog scale (range 0–100, with higher scores indicating more pain) in REDCap. This outcome resulted from a post-intervention survey with children completing a series of visual analog scales after their burn dressing change.

### Statistical analysis

Sample size and power analysis determined the sample size required for our full RCT [8]. We assumed a medium effect size (f2  =  0.15) of the active VR. Using two tails and alpha  =  0.05, a fixed-effect linear regression model would offer power greater than 0.80 with a total sample size of 90 children. We planned to recruit 30 participants for each group to ensure adequate power. Because this study specifically focuses on features of the VR experience, the analysis from the standard-of-care group has been excluded.

Demographic and burn characteristics were described using frequencies and percentages. The Wilcoxon Rank Sum Tests compared the child’s ratings of key VR features (game realism, fun, engagement) between the two VR intervention groups.

Path analysis assessed our hypothesized model of VR features and pain scores. Path analysis, an extension of multiple regression, analyzes theoretically-based relationships [15]. It estimates the magnitude and significance of hypothesized connections between variable sets. This approach lets researchers simultaneously explore direct and indirect effects involving multiple independent and dependent variables. A direct effect is observed when an independent variable directly influences a dependent variable. In contrast, an indirect effect occurs when an independent variable affects a dependent variable through a mediating variable. Standardized path coefficients, which are standardized regression coefficients of VR features in the corresponding path analysis models, describe the direct and indirect effects of VR features on pain.

Path analysis used the Covariance Analysis of Linear Structural Equations procedure from SAS (SAS Institute), which provided path coefficients and significance tests for each path coefficient. Statistical significance was set at α < 0.05, and all tests were two-tailed. SAS version 9.4 conducted data analyses [16].

## Results

### Participants recruited and included in the analysis

Participants were recruited from December 2016 to January 2019. A total of 412 patients were consecutively screened for eligibility with pauses in enrollment due to staff availability, and 240 were deemed potentially eligible and approached (S1 Fig). A total of 95 participants were ultimately recruited. Five participants were excluded because of consent from a non–legally authorized member (n  =  two), issues with the smartphone application (n  =  two), and not completing the study procedures (n = 1). Of the 90 participants, 29 received the standard of care and were excluded from this study. Of the remaining 61 participants, 31 (51%) were assigned to the active VR-PAT and 30 (49%) to the passive VR-PAT.

### Baseline characteristics

Patient demographic and burn characteristics are presented in Table 1. Overall, the mean age of the 61 children was 11.6 years (median, 11.4; min, 6.3; max, 17.8); 29 (48%) were female patients, and 36 (59%) were White patients. Most children had second-degree burns (56 [92%]).

**Table 1:** Baseline demographical characteristics.

Characteristic	Active VR n(%)	Passive VR n(%)	Total n(%)	p-value
Sex				0.89
Female	15 (48%)	14 (47%)	29 (48%)	
Male	16 (52%)	16 (53%)	32 (52%)	
Age				0.12
6–9 years	10 (32%)	11 (37%)	21 (34%)	
10–14 years	13 (42%)	17 (57%)	30 (49%)	
15–17 years	8 (26%)	2 (7%)	10 (16%)	
Race				**0.01**
Caucasian	23 (74%)	13 (43%)	36 (59%)	
African American	3 (10%)	14 (47%)	17 (28%)	
Other	5 (16%)	3 (10%)	8 (13%)	
Total Body Surface Area (TBSA) burned				0.31
Missing	0 (0%)	1 (3%)	1 (2%)	
<1.0%	10 (32%)	13 (43%)	23 (38%)	
1.0–4.9%	19 (61%)	12 (40%)	31 (51%)	
5–25%	2 (7%)	4 (13%)	6 (10%)	
Burn degree				0.36
Missing	1 (3%)	0 (0%)	1 (2%)	
First degree	0 (0%)	0 (0%)	0 (0%)	
Second degree	29 (94%)	27 (90%)	56 (92%)	
Third degree	1 (3%)	3 (10%)	4 (7%)	
Healing degree				**0.02**
Minimal healing	9 (29%)	14 (47%)	23 (38%)	
Partially healed	7 (23%)	10 (33%)	17 (28%)	
Mostly healed	13 (42%)	2 (7%)	15 (25%)	
Completely healed	2 (7%)	4 (13%)	6 (10%)	
Pain medication within 6 hrs				0.98
Missing	1 (3%)	1 (3%)	2 (3%)	
No pain med	19 (61%)	19 (63%)	38 (62%)	
With pain med	11 (35%)	10 (33%)	21 (34%)	

Age, sex, race, TBSA% burn degree, healing degree were from the patient’s record.

Pain medications within 6 hrs were self-reported by the family member or the legal guardian

### Findings for primary outcome

Features of the VR experience have significant direct and indirect effects on self-reported overall pain scores during pediatric burn dressing changes (Table 2). Of the three VR features, engagement is the only one with a significant direct (-0.39) and total (-0.44) effect on self-reported pain score. The direct effect of VR game realism on pain scores was not statistically significant; game realism decreases self-reported pain primarily through an indirect (-0.30) path. Fun presented with a negative direct (-0.18), indirect (-0.03), and total (-0.20) effect on self-reported pain; however, none of these effects were statistically significant.

**Table 2:** Path analysis standardized direct and indirect effects of VR experience on overall pain score (0–100).

Effect	Direct	Indirect	Total
VR realism (0–100)	0.06	-0.30**	-0.23
VR fun (0–100)	-0.18	-0.03	-0.20
VR engagement (0–100)	-0.39**	-0.05	-0.44**
Time thinking of pain during burn dressing (0–100)	0.37**		0.37**

Time spent thinking about pain during the burn dressing was self-reported by patient immediately after the VR intervention using a numerical scale ranging from 0–100, with 0 indicating not thinking about pain at all and 100 indicating thinking about pain the whole time. **P-value <0.05

**Fig 2:**
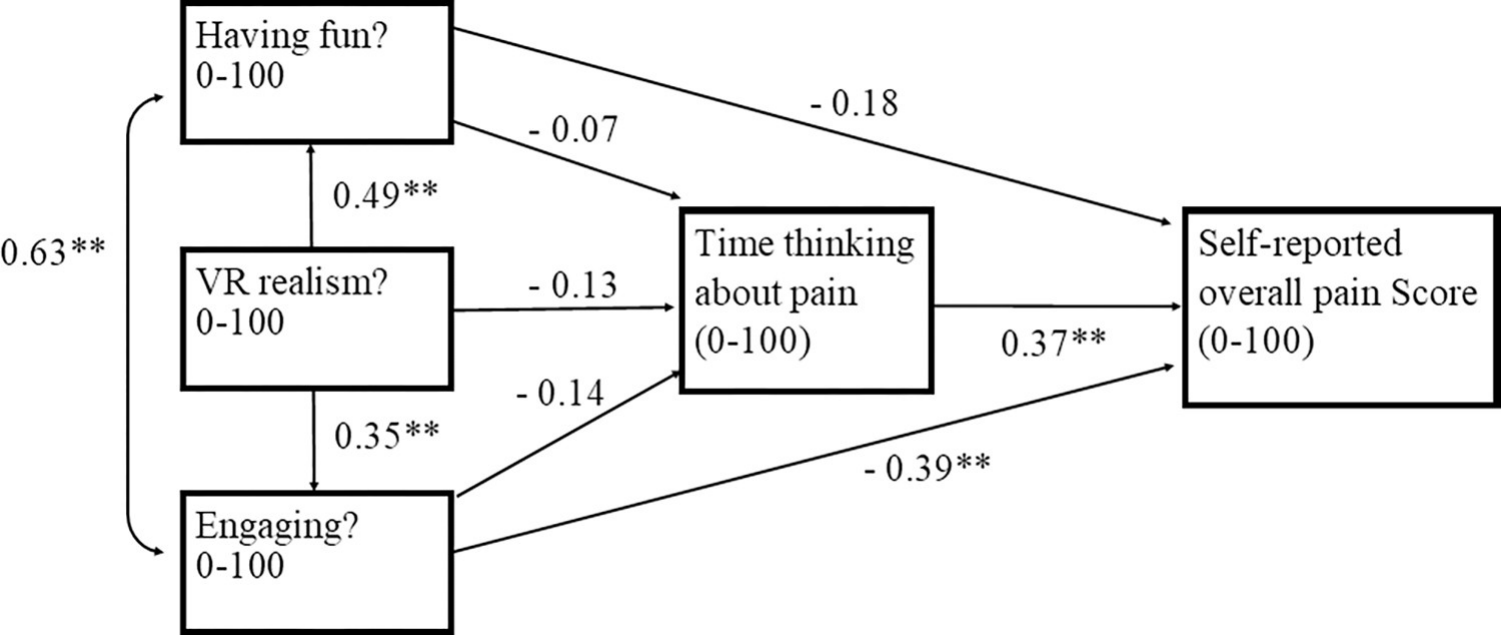
Postulated path causal model for virtual reality pain reduction effect during burn dressing changes. Legend—Association between multiple variables and patient self-reported overall pain is shown by arrows extending from each variables to self-reported overall pain score (0–100). Numbers on each arrow are standardized path coefficients. The higher the coefficient means stronger the association with **indicating a statistical test p-value <0.05. Model fit: X 2 = 17.6, df = 1, p-value = <0.0001; Goodness of Fit Index (GFI) = 0.91.

VR features also exhibit significant interrelationships–a positive bidirectionality between fun and engagement (0.63) and game realism’s positive effect on fun (0.49) and engagement (0.35) (Fig 2).

Time thinking of pain during burn dressing has a significant direct positive effect on self-reported pain (0.37) (Fig 2). All three VR features have a negative direct effect on time thinking of pain during burn dressing (fun, -0.07; game realism, -0.13; engagement, -0.14), although none of the individual path coefficients are statistically significant.

**Fig 3:**
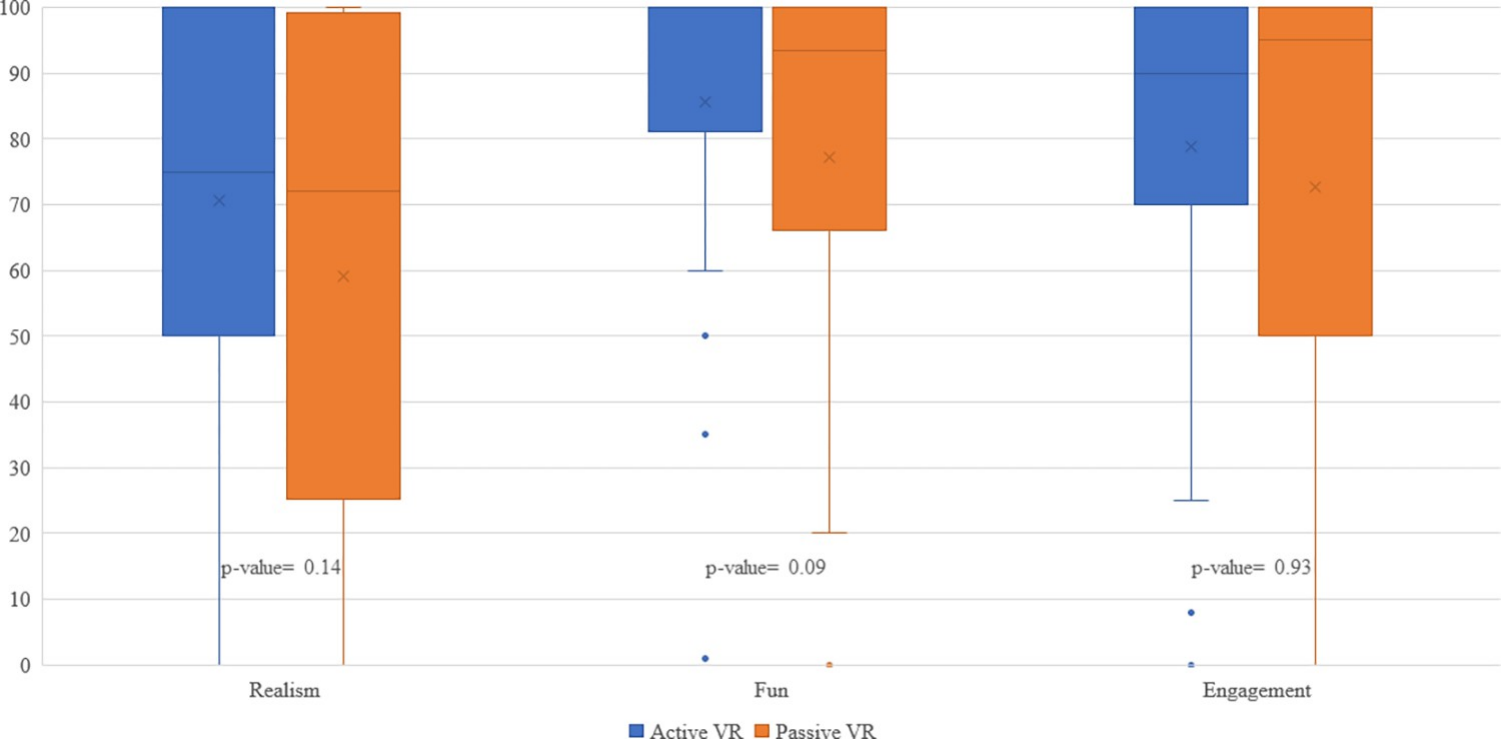
Boxplot of key features of VR experience by active vs. passive VR participants. Legend- Key features of the patient VR experience (game realism, fun, and engagement) reported by patients in the active VR (playing the VR) and passive VR group (immersed in the same VR environment without interactions). P-value was from the nonparametric Wilcoxon rank-sum statistical test comparing the active VR group with the passive VR group.

The difference in median scores of VR features was not statistically significant between the active (game realism, 77.5; fun, 100; engagement, 90) and passive (game realism, 72; fun, 93.5; engagement, 95) VR distraction types (S1 Table). The lower quartiles of VR features in the active VR group were markedly higher than those in the passive VR group. As shown in the box plots for each VR feature, passive VR scores demonstrate a sizeable negative skew (Fig 3).

## Discussion

This study is the first to develop a quantified model of features of the VR experience and their impact on perceived pain. Within the context of VR interventions for procedural pain, the features of the VR experience we included in this study–VR game realism, fun, and engagement–have significant effects on perceiving pain during burn dressing changes. Moreover, our proposed model of VR features, assessed through path analysis, depicts the interrelationships among these features and provides insight into the potential mechanisms through which they influence pain perception.

Prior studies indicate that attentional distraction is the primary mechanism by which VR interventions reduce perceived pain during medical procedures [17,18]. VR serving as a pain alleviation therapeutics is grounded in the capacity theory of attention, which states that humans have limited attentional capacity [19,20]. VR distraction leaves fewer attentional resources for pain processing, thus decreasing the time a patient spends thinking about pain [17]. Our study provides quantitative evidence for distraction as a key mechanism of VR analgesia. To represent the distraction mechanism within our postulated model, the variable “time spent thinking about pain during burn dressing” was included as an intermediary between the VR features and self-reported pain. The path analysis results were consistent with our hypothesized model–each VR feature was negatively associated with time spent thinking about pain which significantly positively influenced self-reported pain. Although none of the individual direct effects of VR features on time spent were statistically significant, collectively, the VR features have a notable impact on time spent. Therefore, children who experienced higher degrees of VR features spent less time thinking about pain during the medical procedure, reducing their perceived pain. Thus, this study adds to the growing body of evidence supporting the effectiveness of the distraction mechanism in VR interventions for pain management.

Although our study provided evidence for the distraction mechanism, our postulated model depicts an additional pathway by which VR reduces perceived pain; VR feature engagement directly reduces self-reported pain. Because this direct path does not operate on time spent thinking of pain, it provides support for an alternative mechanism, beyond mere distraction, by which VR reduces perceived pain. In fact, the magnitude of this direct effect on perceived pain from engagement is greater than that resulting from time spent thinking of pain, suggesting that this additional mechanism is a stronger analgesic mechanism than distraction only. Evidence for mechanisms of VR analgesia for acute pain beyond distraction is limited; however, the direct path from engagement to self-reported pain may represent a neurophysiologic mechanism [21].

A recent study using an electroencephalograph during VR analgesic interventions revealed that active VR decreases the amplitudes of both pre-perceptual and late components of event-related potentials in response to painful stimuli, where the pre-perceptual components relate to early sensory modulation. In contrast, the late elements involve psychologically, emotionally, and cognitively evaluating pain [22]. Researchers attempted to control pain by modulating specific “pain matrix” brain areas. Administering oral morphine among 14 healthy adults modulated the medial frontopolar cortex area (mFP; medial Brodmann area10) [23]. Noninvasive transcranial magnetic stimulation stimulated the prefrontal cortex (PFC) [24] or primary somatosensory cortex (S1) and the second somatosensory cortex (S2) to control pain [25]. Using 15 healthy adult volunteers, VR gaming altered PFC to control thermal pain, in which functional near-infrared spectroscopy captured PFC and S1/S2 signals [26]. Hoffman et al. reported VR could alter brain activities in the anterior cingulate cortex, insula, thalamus, and S1/S2 to generate subjective analgesic effects among nine healthy adult volunteers. Functional magnetic resonance imaging detected brain signals in these brain areas [18]. Our study adds to the limited body of evidence supporting an alternative VR analgesia mechanism beyond distraction. Growing the body of evidence supporting additional mechanisms could offer a compelling rationale to broaden applying VR in pain management, including beyond acute pain settings [27].

Previous studies have named several features or qualities of the VR experience, including presence, interactivity, social interaction, embodiment, immersion, and agency [13,14]. Our study builds upon this work by modeling and quantifying the effects and interrelationships of some of these features. This approach can provide developers of VR analgesia interventions with data to prioritize optimizing certain features. For example, three studies aim to enhance the effectiveness of VR analgesia by maximizing user interactivity and presence in the virtual environment [10–12]. Our path analysis findings strongly support this strategy for reducing perceived pain. Specifically, engagement, which relates to interactivity, directly reduces perceived pain. Similarly realism, which relates to presence, indirectly reduces perceived pain. Our results suggest that further efforts to model and quantify the effects of additional VR features could be highly productive. Importantly, this study contributes a proven approach for future studies to quantify the impact of other potential VR features on pain and thus continue to inform developing optimal VR interventions.

Active VR reduces pain more effectively than passive VR [8]. The differences in VR feature scores between active and passive VR explain some of the differences in effectiveness. Notably, for each VR feature, passive VR presents with a consistently high negative skew, as visualized in the box plots (Fig 3). In other words, for each VR feature, passive VR has many scores far below the median; thus, active VR provides a greater likelihood that users will experience at least close to moderate degrees of game realism, fun, and engagement. The median’s tendency to reduce the influence of skews and extreme values explains the lack of a statistically significant difference in the medians of VR features between the VR groups. The VR features measured in this study likely do not explain the entire difference between the effectiveness of active and passive VR-PAT; several VR features were not included in our research, which probably each accounts for parts of the remaining difference in effectiveness between the VR groups. In future studies, additional VR features would enhance understanding of VR analgesia, inform intervention design, and optimize pain management strategies.

This study has a few limitations that should be addressed in future research. First, this study did not examine the VR interventions over repeated burn dressings. A longitudinal study design would provide valuable information on how the degrees of VR features and their effects on pain change over time. Additionally, there were a couple of significant differences in demographic characteristics between the active and passive VR groups, despite randomization. The passive group had less tissue healing, which may have impacted pain scores. The ethnicities in the active and passive VR group differed significantly, which may also impact pain scores. Another limitation is the subjective nature of the VR feature ratings, as they were self-reported by the children. How children interpreted the concept of each VR feature was potentially variable. For example, children may have interpreted the concept of realism as the similarity of the VR environment to the real world, the sense of embodiment within the VR experience, or the feeling of presence in the virtual environment. Due to the emerging nature of VR research, researchers lack a consensus on terminology. Future studies should consider using specific and clear terminology within the academic community while ensuring the general pediatric population understands the terms. Objective measures of VR features could be developed for future studies to avoid the subjectivity of self-reported scores altogether. A third limitation is that the primary outcome is subjective self-reported overall pain score during the burn dressing changes. While self-reported pain score is widely used as the outcome measurement of pain research around the world, future research needs to develop and test objective responsive neuroimaging biomarker(s) for clinical trials of VR pain alleviation therapeutics.

In conclusion, our findings suggest that three features of the VR experience (game realism, fun, and engagement) may indirectly influence pain perception during burn dressing changes at the pediatric outpatient burn clinic. Among these features, engagement emerges as a significant direct influencer of pain perception. Our model of these interrelated features provides insight into the potential mechanisms through which VR affects pain perception, supporting distraction as a key mechanism of VR analgesia and highlighting an alternative, potentially neurophysiological, mechanism beyond mere distraction. Modeling and quantifying additional VR features would inform developing more effective VR interventions. Future studies should employ longitudinal designs, develop objective measures of VR features, develop methods to manipulate VR features, consider clear and specific terminology to describe digital experiences, and expand to other pediatric medical care procedures such as acute pain management during emergency department visits, orthopedic care, and laser dermatology treatment, etc.

## Supporting information

S1 Fig Trial profile: CONSORT Flow Diagram. (TIF)

S1 Table Mean and median of self-reported VR experience. (DOCX)

S1 Appendix CONSORT Checklist. (DOCX)
