# Supplementary figures and images for: Features of virtual reality impact effectiveness of VR pain alleviation therapeutics in pediatric burn patients: A randomized clinical trial
Source: PLOS Digit Health. 2024 Jan 25;3(1):e0000440. doi: 10.1371/journal.pdig.0000440 (PMC10810440; doi:10.1371/journal.pdig.0000440)

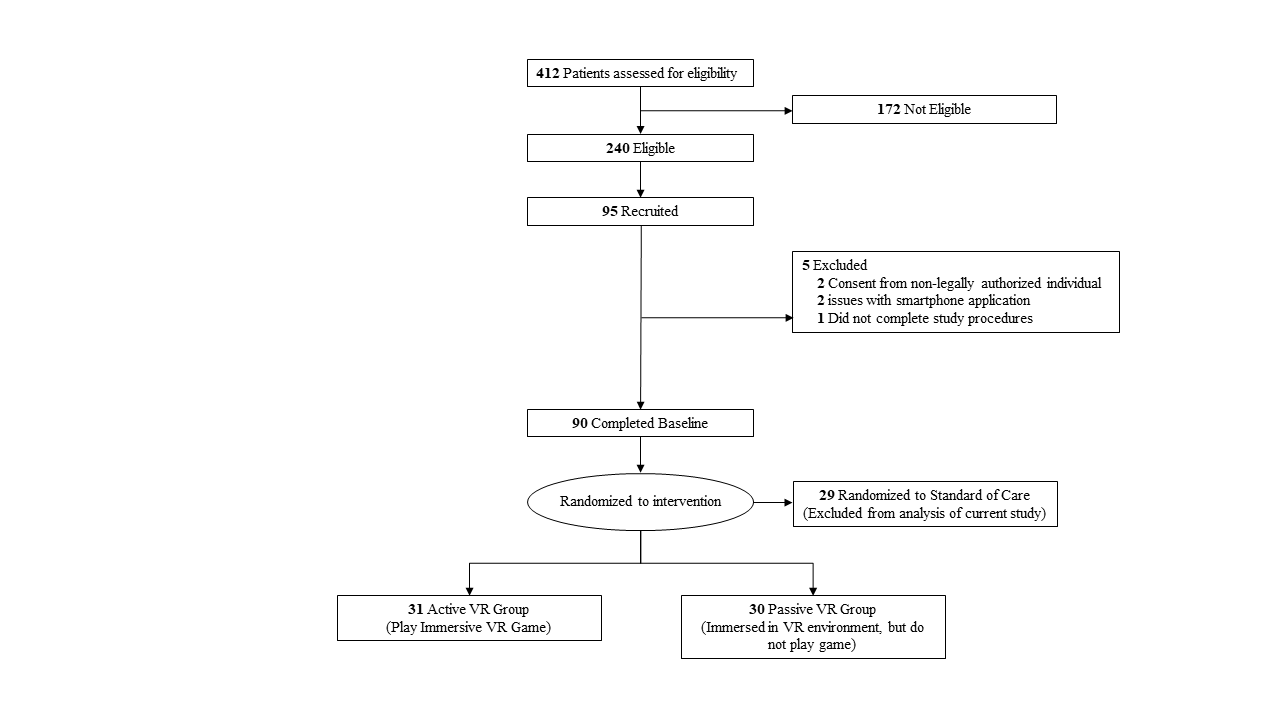

Supplement: S1 Fig — (TIF) [file pdig.0000440.s001.tif]
