# Supplementary material for: Features of virtual reality impact effectiveness of VR pain alleviation therapeutics in pediatric burn patients: A randomized clinical trial
Source: PLOS Digit Health. 2024 Jan 25;3(1):e0000440. doi: 10.1371/journal.pdig.0000440 (PMC10810440; doi:10.1371/journal.pdig.0000440)
Supplement: S1 Table — (DOCX) [file pdig.0000440.s002.docx]

**S1 Table. Mean and median of self-reported VR experience**

| **Feature** | **Distraction Type** | **Mean** | **Median** | **Lower Quartile** | **Upper Quartile** | **P-Value** |
| --- | --- | --- | --- | --- | --- | --- |
| VR Realism (numerical scale 0-100) |  |  |  |  |  | 0.14 |
|  | Active VR (n=31) | 73.1 | 77.5 | 50.0 | 100.0 |  |
|  | Passive VR (n=30) | 59.1 | 72.0 | 29.0 | 99.0 |  |
| VR Fun (numerical scale 0-100) |  |  |  |  |  | 0.09 |
|  | Active VR (n=31) | 85.7 | 100.0 | 81.0 | 100.0 |  |
|  | Passive VR (n=30) | 77.3 | 93.5 | 68.0 | 100.0 |  |
| VR Engagement (numerical scale 0-100) |  |  |  |  |  | 0.93 |
|  | Active VR (n=31) | 78.9 | 90.0 | 70.0 | 100.0 |  |
|  | Passive VR (n=30) | 72.7 | 95.0 | 50.0 | 100.0 |  |
| VR realism, fun, and engagement were patient's self-reported VR experience using the numerical scale 0-100, with 0 indicating least experience and 100 indicating the maximum experience.  P-value was from the nonparametric Wilcoxon rank-sum test comparing active VR group with passive VR group | | | | | | |
